# Haplodeficiency of Ataxia Telangiectasia Mutated Accelerates Heart Failure After Myocardial Infarction

**DOI:** 10.1161/JAHA.117.006349

**Published:** 2017-07-19

**Authors:** Lixin Jia, Wenmei Zhang, Youcai Ma, Boya Chen, Yan Liu, Chunmei Piao, Yuan Wang, Min Yang, Tingting Liu, Junmeng Zhang, Taotao Li, Shaoping Nie, Jie Du

**Affiliations:** ^1^ Beijing Anzhen Hospital Capital Medical University Beijing Institute of Heart, Lung and Blood Vessel Diseases Beijing China; ^2^ The Collaborative Innovation Center for Cardiovascular Disorders Beijing Institute of Heart, Lung and Blood Vessel Diseases Beijing China; ^3^ The Key Laboratory of Remodeling‐Related Cardiovascular Diseases Capital Medical University Ministry of Education Beijing Institute of Heart, Lung and Blood Vessel Diseases Beijing China; ^4^ Emergency & Critical Care Center Beijing Anzhen Hospital Capital Medical University Beijing China

**Keywords:** angiogenesis, fibroblasts, myocardial infarction, senescence, vascular endothelial growth factor, Animal Models of Human Disease, Basic Science Research, Fibrosis, Myocardial Infarction, Remodeling

## Abstract

**Background:**

Cell senescence is involved in the process of organ damage and repair; however, the underlying molecular mechanism needs to be further explored.

**Methods and Results:**

Senescence‐related genes (ie, p21, p53, and ataxia telangiectasia mutated [ATM]) were shown to be elevated after myocardial infarction (MI) in both mouse and human hearts. Ten‐ to 12‐week‐old male wild‐type littermates (ATM ^+/+^) and ATM heterozygous mice (ATM ^+/−^) were subjected to MI. Cardiac echography showed that ATM haplodeficiency did not affect the survival rate but aggravated heart failure at day 28 post MI. Histologic analysis showed increased fibrosis in the noninfarct area of ATM ^+/−^ mice compared with that in ATM ^+/+^ mice. Senescence‐associated β‐galactosidase staining showed that the number of senescent fibroblasts was decreased when ATM was haplodeficient both in vivo and in vitro. Costaining of α‐smooth muscle actin with p53 or p19 showed fewer senescent myofibroblasts in ATM ^+/−^ mouse hearts. Moreover, angiogenesis was also examined using the endothelial markers CD31 both at early (day 7) and late stages (day 28) after MI, and ATM haplodeficiency reduced angiogenesis after MI. Finally, cardiac fibroblasts were isolated from infarcted mouse heart and the medium were tested for its capacity of endothelial tubing formation, revealing that ATM haplodeficiency led to lower vascular endothelial growth factor production from cardiac fibroblast and reduced capacity of endothelial tube formation in vitro.

**Conclusions:**

The present study shows that ATM haplodeficiency decreases fibroblast senescence and vascular endothelial growth factor production and impaired angiogenesis in response to MI, leading to accelerated heart failure.

**Clinical Perspective**

**What Is New?**

- Myocardial infarction induces cardiac fibroblast senescence and vascular endothelial growth factor production in an ataxia telangiectasia mutated–dependent manner, which improves collateral angiogenesis and preserves heart function.

**What Are the Clinical Implications?**

- Identifying molecules that regulate fibroblast senescence may represent a novel therapeutic approach to improve heart function post myocardial infarction.

## Introduction

Myocardial infarction (MI) results in complex architectural myocardial alteration and leads to heart failure.^1^ Apoptosis and necrosis of cardiomyocytes in response to infarction triggers inflammation and an imbalance of collagen synthesis and degradation, in which cardiac fibroblasts are also involved.^2^, ^3^ Activated myofibroblasts in the infarcted heart migrate into the injured myocardium, replace the damaged cardiomyocytes, and form scar tissue to avoid cardiac rupture. However, the excessive myofibroblast accumulation and fibrosis in the uninjured area of the heart contributes to adverse cardiac remodeling and heart failure.^3^ Moreover, the balance of the proinflammatory and anti‐inflammatory environment, as well as angiogenesis post MI, also play key roles in regulating cardiac remodeling and function. Therefore, a better understanding of the underlying molecular and cellular mechanisms involved in this process is critical for preserving heart function post MI.

Cellular senescence has recently been reported to limit organ fibrosis via regulating the fate of fibroblasts.^4^ Senescence is induced by various stimuli in both physiological and pathological processes.^5^ Although senescent cells exhibit growth arrest and function loss, they are metabolically active and secret multiple proteins, known as senescence‐associated secretory phenotype (SASP).^6^, ^7^ SASP contains a large number of cytokines and chemokines, which exert roles in regulating inflammation and angiogenesis. In addition, we previously observed that cardiac fibroblast senescence was involved in cardiac remodeling after MI,^8^ but the molecular mechanisms and how fibroblast senescence contributed to post‐MI cardiac remodeling remains unclear. Among multiple signaling pathways involved in senescence, p53‐mediated signaling pathways are closely associated with post‐MI cardiac remodeling and function through affecting cardiomyocyte apoptosis,^9^, ^10^ as well as angiogenesis.^11^

It is known that p53 can be activated by multiple stimuli and upstream signaling pathways. As an upstream signaling protein of p53, ataxia telangiectasia mutated (ATM) activates p53 by selective phosphorylation, thereby regulating p53‐dependent signaling pathway^12^ and cell cycle checkpoints.^13^ ATM is required for DNA repair and maintenance of genomic homeostasis.^14^ ATM induces cell senescence in an ATM/p53–dependent signaling pathway in tumor cells^15^ and endothelial cells.^16^ ATM can be activated by DNA double‐strand breaks and oxidative stress,^17^ which are involved in MI^18^, ^19^ as well as post‐MI cardiac remodeling.^19^

In the present study, we investigated the role of cellular senescence in post‐MI cardiac remodeling and heart failure as well as the underlying mechanisms. ATM knockout mice were first reported in 1996.^20^ While ATM‐deficient mice are viable, their growth is retarded and are infertile. However, the ATM haplodeficient mice display no abnormalities through 8 months of age from the wild‐type (WT) littermate. To avoid the development of retardation in cardiac remodeling, ATM‐haplodeficient mice were used in our study. We demonstrate the loss of senescence in worsening cardiac injury, angiogenesis, and heart dysfunction in a haplodeficiency model of ATM, an upstream signaling protein of p53, in MI‐induced cardiac injury. Mechanistically, ATM regulates a cardiac fibroblast senescence phenotype and production of vascular endothelial growth factor (VEGF), which, in turn, regulates collateral angiogenesis, preserving cardiac function.

## Methods

### Animal Model

WT littermates (ATM^+/+^) and ATM heterozygous (ATM^+/−^) mice on a C57BL/6 background were obtained from the Jackson Laboratory. A total of 93 ATM^+/+^ and 102 ATM^+/−^ male mice (10–12 weeks old) were used in the present study. A total of 15 ATM^+/+^ and 15 ATM^+/−^ mice underwent sham surgery, while 78 ATM^+/+^ and 87 ATM^+/−^ mice underwent MI surgery. To induce the MI model, mice were anesthetized with a mixture of 1% to 2% isoflurane and oxygen inhalation and subjected to our surgical MI model by ligation of the left coronary artery, as previously described.^8^ The sham group underwent the thoracotomy procedure, but no further suture was made to the left coronary artery. Buprenorphine (0.05 mg/kg per 12 hours, IP) was administrated for 48 hours after surgery to ease postoperative analgesia. Mice from both the MI and sham groups were euthanized by CO_2_ inhalation at the indicated time.^8^, ^21^ All animal protocols were approved by the Animal Care and Use Committee of Capital Medical University. The investigation conforms to the Guide for the Care and Use of Laboratory Animals published by the US National Institutes of Health (NIH Publication No. 85‐23, revised 2011).

### Echocardiography

Echocardiography was performed on a Vevo 770 High‐Resolution Imaging System (Visual Sonics, Inc) with a 15‐MHz ultrasound probe. Mice were anesthetized in a chamber with a mixture of 4.0% to 5.0% isoflurane and oxygen, and maintained by a mixture of 1.0% to 2.0% isoflurane and oxygen. Parasternal long‐axis and midventricular short‐axis views were obtained in the 2‐dimensional mode, and M‐mode tracings at the papillary muscle level were recorded.

### Histology and Immunohistochemistry

Both heart tissues were fixed in 10% paraformaldehyde, embedded in paraffin, and sectioned at 5‐μm intervals. Masson's trichrome staining and picrosirius red staining were performed using standard procedures as described elsewhere.^22^, ^23^ Computerized planimetry of the histological images of the stained sections was used to measure and calculate: (1) scar thickness (average of 5 equidistant measurements) and septum thickness (average of 3 equidistant measurements); (2) epicardial and endocardial circumference and circumference occupied by infarcted wall (infarct size expressed as percentage of total left ventricular circumference)^24^; and (3) expansion index, as defined by Hochman and Choo,^25^ which is expressed as (left ventricular cavity area/total left ventricular area)×(septum thickness/scar thickness). Fibrosis was quantitated by measuring the total blue area (mm^2^) with a NIS‐ELEMENTS quantitative automatic program (Nikon) in the Masson's trichrome–stained heart sections.^23^

Immunohistochemical staining was performed as described elsewhere.^26^ Briefly, heart sections (5 μm) from paraffin‐embedded tissues were deparaffinized and incubated with anti‐mouse monoclonal antibodies against α‐smooth muscle actin (α‐SMA) (1:200 dilution, Santa Cruz Biotechnology) and CD31 (1:200 dilution, Cell Signaling Technology) at 4°C overnight. The sections were then incubated with a biotinylated secondary antibody for 30 minutes at 37°C.

For immunofluorescent staining, cryosections were incubated with primary antibodies for p19 (1:50 dilution, Abcam), p53 (1:50 dilution, Cell Signaling Technology), α‐SMA (1:200 dilution), Collagen I (1:100 dilution, Rockland Immunochemicals), and IgG at 4°C overnight, then with fluorescein isothiocyanate– or tetramethylrhodamine‐conjugated secondary antibodies (Jackson ImmunoResearch) at room temperature for 1 hour. All sections were mounted with DAPI (Invitrogen). Sections were viewed under a 2‐photon laser scanning microscope (TCS 4D; Leica). A commercial kit DeadEnd Fluorometric Tunel (Promega) was used to detect cell apoptosis.

The senescence‐associated β‐galactosidase (SA‐β gal) activity assay in cardiac sections or cultured cardiac fibroblasts was performed, as previously described.^8^, ^27^ For cryostat sections, tissue was embedded in O.C.T. Compound (Sakura Finetek), frozen in liquid nitrogen, and stored at −80°C until sectioning.

### RNA Extraction and Real‐Time Polymerase Chain Reaction Analysis

Total RNAs were extracted using the Trizol method according to the manufacturer's protocol (Invitrogen). Two micrograms of purified RNA were used for cDNA synthesis. Reverse transcription of mRNA was accomplished by 2x SYBR Master Mix (Takara), using a BIO‐RAD iCycler iQ5 (Bio‐Rad). Primers used in this study were as follows: ATM, forward 5′‐GCTGCCATACTTGATCCATG‐3′ and reverse TCCGAATTTGCAGGAGTTG; p53, forward 5′‐GTCGTACCCCGATTCAGGTG‐3′ and reverse 5′‐TCTGCACCGTAGTTGAGCAG‐3′; p21, forward 5′‐TTGTCGCTGTCTTGCACTCT‐3′ and reverse 5′‐TTTCGGCCCTGAGATGTTCC‐3′; α‐SMA, forward 5′‐ACTCTCTTCCAGCCATCTTTCA‐3′ and reverse 5′‐ATAGGTGGTTTCGTGGATGC‐3′; collagen I, forward 5′‐GAGCGGAGAGTACTGGATCG‐3′ and reverse 5′‐TACTCGAACGGGAATCCATC‐3′; collagen III, forward 5′‐TCCCCTGGAATCTGTGAATC‐3′ and reverse 5′‐TGAGTCGAATTGGGGAGAAT‐3′; Cxcl1, forward 5′‐ACTCAAGAATGGTCGCGAGG‐3′ and reverse 5′‐ GTGCCATCAGAGCAGTCTGT‐3′; Cxcl12, forward 5′‐GGTGCTCAAACCTGACGGTAA‐3′ and reverse 5′‐CAGACAGAGCAGGGCCTTAT‐3′; Cxcl8, forward 5′‐CAGAGACAGCAGAGCACACA‐3′ and reverse 5′‐GATGGTTCCTTCCGGTGGTT‐3′; VEGF, forward 5′‐GCAGCGACAAGGCAGACTAT‐3′ and reverse 5′‐AATCCCAGAGCACAGACTCC‐3′; and GAPDH, forward 5′‐CCTGGAGAAACCTGCCAAGTATGA‐3′ and reverse 5′‐TTGAAGTCACAGGAGACAACCTGG‐3′. Real‐time polymerase chain reaction (PCR) was performed using the Bio‐Rad iQ5 (Hercules).

### Western Blot Analysis

Mouse hearts after sham surgery or MI injury were harvested at the indicated time points and divided into infarcted and noninfarcted parts. Samples were lysed and separated by 8% SDS‐PAGE. The blot was then probed with primary antibodies: anti‐GAPDH (1:2000 dilution), anti–α‐SMA (1:1000 dilution), anti‐p21 (1:1000 dilution), anti‐CD31 (1:1000 dilution), and anti‐VE‐cadherin (1:1000 dilution, Santa Cruz Biotechnology) and anti‐VEGF (1:1000 dilution, Santa Cruz Biotechnology) primary antibodies, and then with infrared dye–conjugated secondary antibodies (1:5000, Rockland Immunochemicals Inc) for 1 hour. Blots were then exposed and analyzed with the Odyssey Infrared Imaging System (LI‐COR Biosciences).

### Cardiac Fibroblast Isolation and Cell Culture

Cardiac fibroblasts were isolated and cultured as described elsewhere.^8^, ^28^ Briefly, hearts from ATM^+/+^ and ATM^+/−^ mice (1–2 days old) were minced and digested. The supernatant was filtered through a 400‐μm nylon mesh filter, followed by centrifugation at 300*g* for 5 minutes. Cells were then placed into dishes with fibroblast culture media (DMEM, 10% normal bovine serum albumin, and 1% penicillin/streptomycin) and incubated at 37°C with 5% CO_2_ and 95% oxygen. Hypoxia/oxygenation was induced as previously described.^8^

Cardiac fibroblast from post‐MI heart were isolated and cultured as previously described.^29^ In brief, hearts from ATM^+/+^ and ATM mice 3 days post MI were removed, minced, and digested with 0.2% collagenase II at 37°C for 30 minutes and dispase at 37°C for 1 hour. Then, cells were placed into dishes with fibroblast culture media. The fibroblast culture medium was collected and concentrated by ultracentrifugation method.

For tube formation analysis, human umbilical vein endothelial cells were seeded on Matrigel‐covered dishes as described^30^ and cultured with extracellular matrix (10% normal bovine serum albumin, and 1% penicillin/streptomycin and 1% endothelial cell growth supplement) containing concentrated fibroblast culture medium.

### Statistical Analysis

Data are presented as means±SEMs. Data were analyzed using nonparametric testing (Mann–Whitney *U* test for 2 groups and Krushal–Wallis *H* test for >2 groups). Repeated‐measures analysis was used to evaluate the statistical significance of data acquired from the same animal over time. *P*<0.05 was considered statistically significant. All statistical analyses were performed with GraphPad software (GraphPad Prism version 5.00 for Windows).

Additional information of methods used in the present study is available in Data S1.

## Results

### Senescence is Involved in Post‐MI Remodeling Both in Human and Mouse Hearts

**Figure 1:**
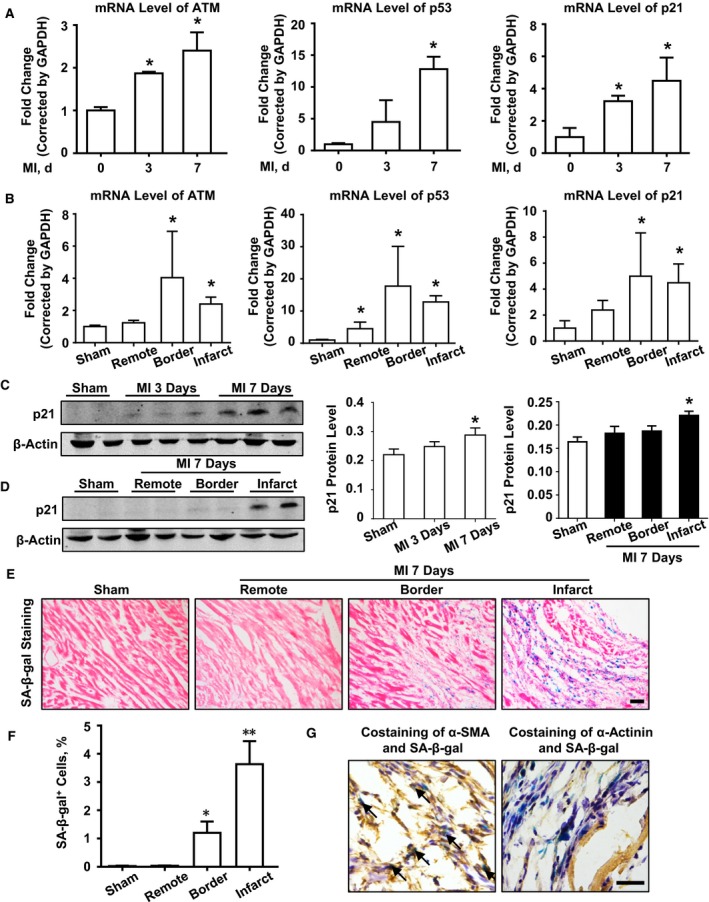
Senescence is involved in post–myocardial infarction (MI) cardiac remodeling in mouse heart. A, Real‐time polymerase chain reaction (PCR) analysis of ataxia telangiectasia mutated (ATM), p53, and p21 mRNA levels at days 0, 3, and 7 after MI. B, Real‐time PCR analysis of ATM, p53, and p21 mRNA levels in sham mouse and different regions (remote area, border area, and infarct area) at day 7 post MI. n=4 in each group. *P<0.05 vs the day 0 or sham group. Western blot analysis and bar graph of (C) p21 expression in wild‐type mouse hearts at indicated times after sham or MI surgery, and (D) p21 expression in different regions of wild‐type mouse hearts 7 days after sham or MI surgery. E, Representative images and (F) statistical analysis of senescence‐associated β‐galactosidase (SA‐β‐gal) staining in mouse hearts at day 7 after sham or MI surgery. n=4 in each group. *P<0.05 vs the sham group, **P<0.01 vs the sham group. G, Representative images of costaining of SA‐β‐gal and α‐smooth muscle actin (α‐SMA)/α‐actinin staining in heart section at day 7 after MI surgery. Bar=50 μm.

**Figure 2:**
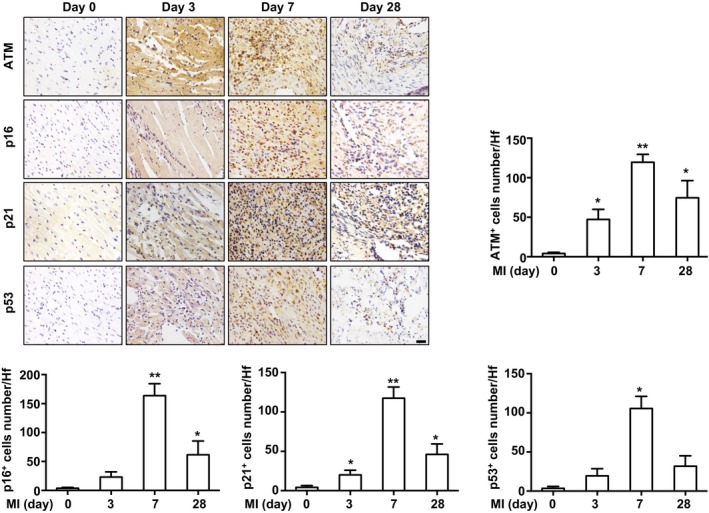
Senescence‐associated gene expression in infarcted mouse heart. Immunostaining and bar graph of senescent‐related proteins ataxia telangiectasia mutated (ATM), p16, p21, and p53 expression in infarcted mouse heart at the indicated time (infarct area). n=5 in each group. *P<0.05, **P<0.01 vs the day 0 group. Bar=50 μm. MI indicates myocardial infarction; HF, high‐power field.

We first examined senescence‐related gene expression in mouse heart after undergoing either sham or MI surgery. Real‐time PCR analysis showed that the mRNA levels of senescence‐related genes, including p21, p19, p53, and ATM, were upregulated in mouse heart post MI (Figure 1A). These proteins were mainly expressed in the infarct and border areas at day 7 post MI (Figure 1B). Furthermore, Western blot analysis of p21, the senescence‐related protein, was measured in post‐MI mouse heart at the indicated time points to show it was upregulated at day 7 post MI, especially in the infarcted region (Figure 1C and 1D). Then, SA‐β‐gal staining was performed to demonstrate that there were significantly increased SA‐β‐gal–positive cells in both infarct and border regions in mouse heart 7 days post MI (Figure 1E and 1F). To identify the cellular type of senescent cells, costaining of SA‐β‐gal and α‐SMA/α‐actinin was performed to show that double positive cells in infarcted mouse heart was mostly fibroblast and not cardiomyocyte (Figure 1G). To examine the role of senescence in acute and chronic phases during post‐MI remodeling, we performed immunohistochemical staining of senescence‐related gene (ie, ATM, p53, p21, and p16) expression at days 0, 3, 7, and 28 post MI. As shown in Figure 2, MI increased senescence‐related gene expression in infarcted mouse heart, especially in the acute phase. In addition, the elevated levels of senescence‐related protein ATM, p16, p21, and p53 were confirmed in the normal and infarcted human hearts (Figure S1).

### ATM Haplodeficiency Aggravates Post‐MI Cardiac Dysfunction

**Figure 3:**
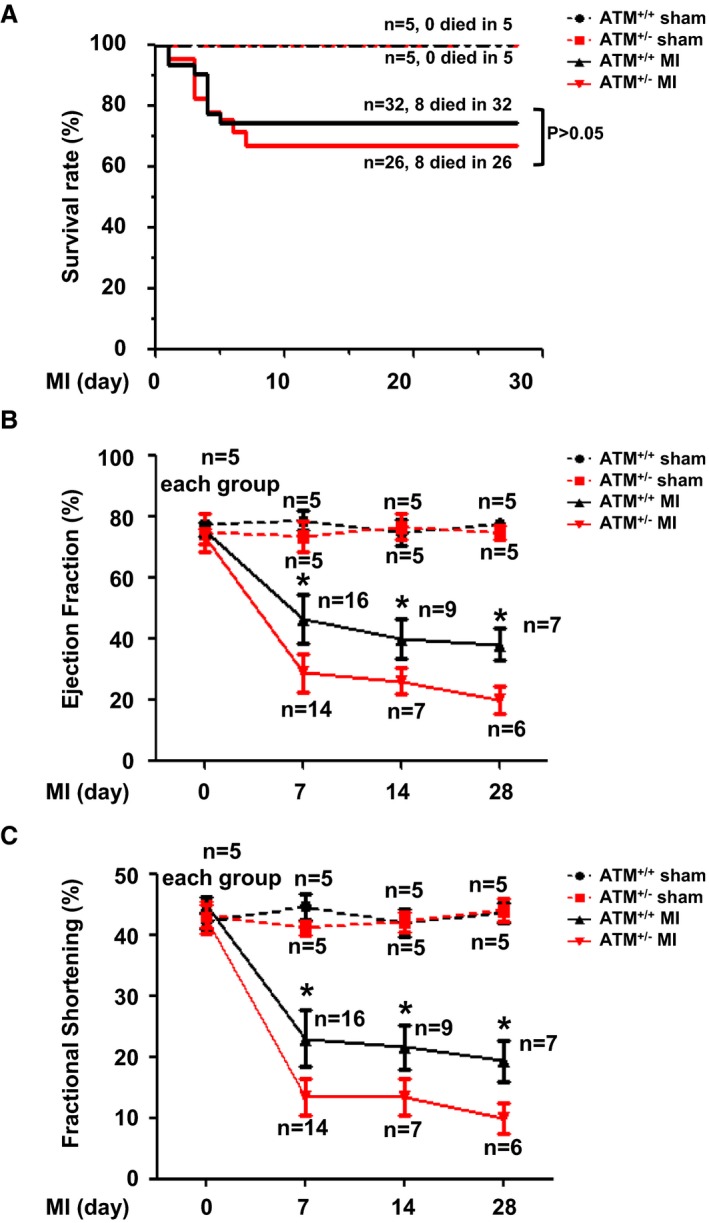
Ataxia telangiectasia mutated (ATM) haplodeficiency aggravated cardiac dysfunction post myocardial infarction (MI). A, Survival rates were analyzed in ATM ^+/+^ and ATM ^+/−^ mice after sham or MI surgery. Echocardiography was performed to show (B) ejection fraction and (C) fraction shortening change in ATM ^+/+^ and ATM ^+/−^ mice after sham or MI surgery. *P<0.05 vs the ATM ^+/−^ MI group.

To explore the role of senescence in post‐MI cardiac remolding, we used mice haplodeficient in ATM, an important senescence effector, and compared the cardiac structure and function with that of WT mice after MI surgery. 2,3,5‐Triphenyltetrazolium chloride staining was performed at 24 hours post MI in ATM^+/+^ and ATM^+/−^ mice, and there was no difference in the infarct area between 2 groups (Figure S2A). Indices of left ventricular function were assessed by echocardiography at baseline and at days 0, 7, 14, and 28 post MI. MI caused significantly more deaths and the survival rate was recorded up to 28 days after surgery. Mice that died within 24 hours were excluded. ATM haplodeficiency did not affect the survival rate compared with that of WT mice after MI surgery (Figure 3A). As shown in Figure 3B and 3C, baseline left ventricular function and geometry were not different between ATM^+/+^ and ATM^+/−^ mice, but ATM haplodeficiency exacerbated post‐MI cardiac dysfunction from day 7 post MI. Echocardiographic measurement at day 28 post MI revealed acceleration of cardiac dilation and deterioration of left ventricular function in ATM^+/−^ mice compared with control mice (Table). Furthermore, both the heart and lung weights adjusted for body weight were higher than those of WT mice in response to MI.

**Table 1:** Body and Organ Weights and Echocardiographic Data 28 Days Post MI

	Sham		MI	
	ATM^+/+^ (n=4)	ATM^+/−^ (n=4)	ATM^+/+^ (n=7)	ATM^+/−^ (n=6)
No.	3	3	11	12
BW, g	28.75±0.79	27.32±0.88	25.49±1.72	26.15±0.85
HW/BW	5.20±0.25	5.40±0.28	7.47±1.01^a^	8.52±1.28^a^, ^b^
LW/BW	4.95±0.13	4.82±0.09	6.38±0.62^a^	7.62±0.83^a^, ^b^
IVSd	0.86±0.09	0.85±0.14	0.81±0.11	0.49±0.20^a^
IVSs	1.52±0.08	1.45±0.06	1.15±0.19	0.59±0.27^a^, ^b^
LVIDd	3.44±0.02	3.41±0.16	4.98±0.84^a^	5.85±1.67^a^, ^b^
LVIDs	1.91±0.05	1.96±0.28	4.16±1.11^a^	5.39±1.95
LVPWd	0.85±0.08	0.78±0.02	0.58±0.20	0.55±0.31
LVPWs	1.40±0.11	1.28±0.08	0.83±0.35^a^	0.73±0.48^a^, ^b^
EF, %	77.16±1.66	74.55±2.21	38.18±4.75^a^	20.83±4.36^a^, ^b^
FS, %	44.82±1.26	42.58±3.35	19.34±3.91^a^	9.89±2.01^a^, ^b^
LV mass (corrected)	81.26±0.07	74.47±2.25	103.01±18.74^a^	94.16±31.13^a^, ^b^

Values are mean±SEM. ATM indicates ataxia telangiectasia mutated; BW, body weight; EF, ejection fraction; FS, fractional shortening; HW, heart weight; IVSd, interventricular septum in diastole; IVSs, interventricular septum in systole; LV, left ventricular; LVIDd, left ventricular internal diameter in diastole; LVIDs, left ventricular internal diameter in systole; LVPWd, left ventricular posterior wall in diastole; LVPWs, left ventricular posterior wall in systole; LW, lung weight.

a *P*<0.05 vs the corresponding sham group.

b *P*<0.05 vs the control myocardial infarction (MI) group.

### ATM Haplodeficiency Deteriorates Post‐MI Cardiac Remodeling

**Figure 4:**
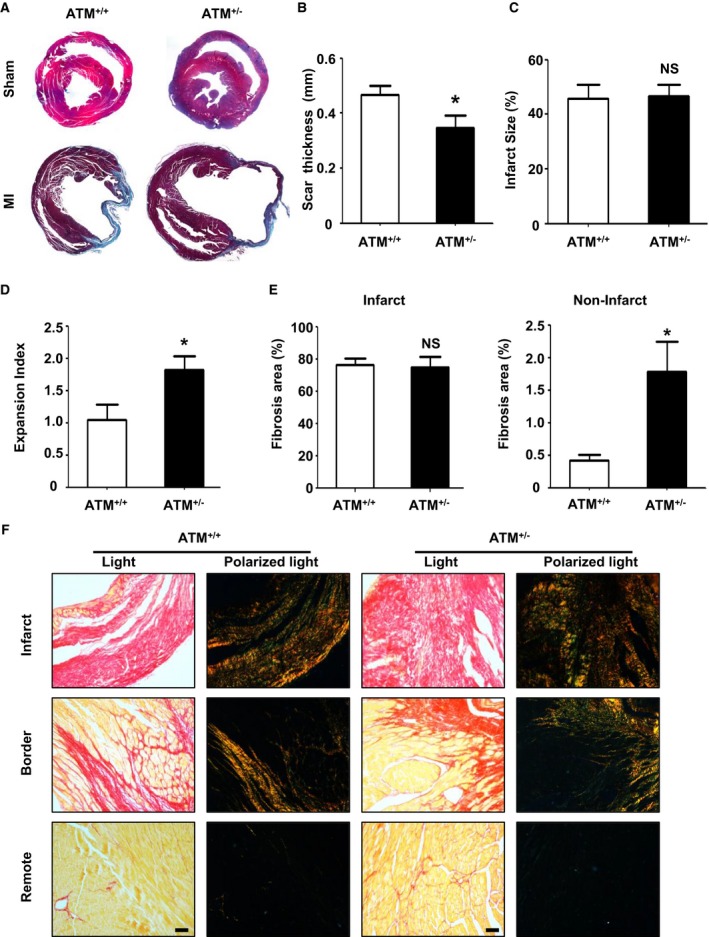
Ataxia telangiectasia mutated (ATM) haplodeficiency increased cardiac fibrosis post myocardial infarction (MI). A, Representative Masson's trichrome staining of cardiac sections in ATM ^+/+^ and ATM ^+/−^ mice at day 28 after sham or MI surgery. B, Wall thickness of scar tissue and quantitative analysis of (C) infarct size at day 28 after MI in ATM ^+/+^ and ATM ^+/−^ mice. D, Expansion index of left ventricle at 28 days after MI in ATM ^+/+^ and ATM ^+/−^ mice. E, Quantification of fibrosis in infarcted and noninfarcted areas 28 days after MI in ATM ^+/+^ and ATM ^+/−^ mice. F, Representative picrosirius red–stained sections of infarct, border, and remote areas 28 days after MI in ATM ^+/+^ and ATM ^+/−^ mice. n=7 in the ATM ^+/+^ MI group, n=6 in the ATM ^+/−^ group. *P<0.05 vs the ATM ^+/+^ MI group. NS indicates not significant. Bar=50 μm.

Cardiac remodeling is associated with alterations in the geometric characteristics of the ventricle, dilation, and hypertrophy.^31^ Cardiac remodeling was analyzed 28 days after MI to determine the long‐term role of ATM in post‐MI remodeling. Results from Masson's trichrome staining revealed similar gross morphology in sham‐operated hearts between ATM^+/+^ and ATM^+/−^ mice, whereas ATM‐haplodeficient mice demonstrated enhanced wall thinning and expansion compared with the control group without affecting scar formation (Figure 4A through 4D). Picrosirius red staining was also performed, and the fibrotic area was increased in the ATM^+/−^ group in the noninfarcted area 28 days after MI (Figure 4E and 4F). To assess the influence of ATM haplodeficiency on post‐MI cardiac hypertrophy, we also performed wheat germ agglutinin staining, and the cross‐sectional cardiomyocyte area in ATM^+/−^ mice was markedly larger than that in WT mice 28 days after MI (Figure S2B) and showed disarrayed collagen fiber alignment within the infarct zone in ATM^+/−^ mice after MI (Figure S2C). It is known that ATM regulates cell apoptosis, which is also important for post‐MI remodeling. We therefore examined whether there was any difference in cardiomyocyte apoptosis, as shown in Figure S1D. Results from terminal deoxynucleotidyl transferase dUTP nick end labeling staining showed no difference on cell apoptosis at days 1 and 3 post MI, but ATM haplodeficiency exhibited an increase in apoptosis at day 7 post MI (Figure S2D).

### ATM Haplodeficiency Increases Myofibroblast Accumulation Through Inhibiting Senescence

**Figure 5:**
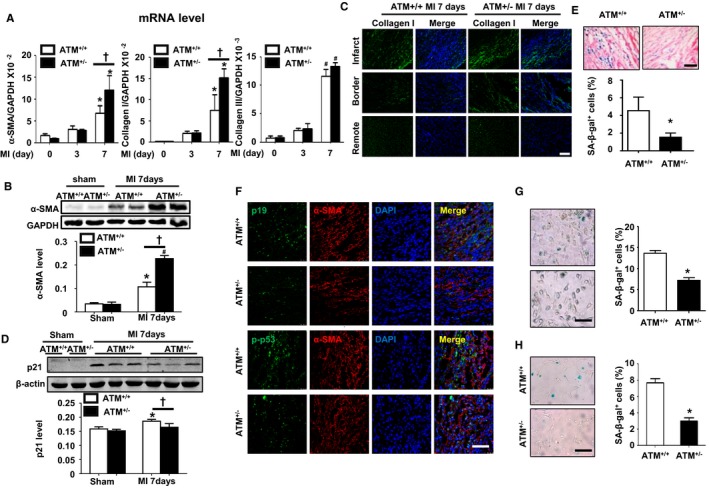
Ataxia telangiectasia mutated (ATM) haplodeficiency increases myofibroblast accumulation and decreases fibroblast senescence both in vivo and in vitro. A, Quantitative real‐time polymerase chain reaction (PCR) was performed to detect the mRNA level of α‐smooth muscle actin (α‐SMA), collagen I, and collagen III in the hearts of ATM ^+/+^ and ATM ^+/−^ mice at the indicated time points post‐myocardial infarction (MI). n=4 in each group. *P<0.05, ^#^ *P*<0.01 vs the corresponding sham group; ^†^ *P*<0.05 vs the control MI group. B, Western blot analysis of α‐SMA levels in the hearts of ATM ^+/+^ and ATM ^+/−^ mice at the indicated time points post MI. C, Representative collagen I–stained sections of infarct, border, and remote size 7 days after MI in ATM ^+/+^ and ATM ^+/−^ mice. D, Western blot analysis of p21 levels in the hearts of ATM ^+/+^ and ATM ^+/−^ mice at the indicated time points post MI. E, Representative images and statistical analysis of senescence‐associated β‐galactosidase (SA‐β‐gal) staining in cardiac sections from ATM ^+/+^ and ATM ^+/−^ mice after MI. F, Representative images of costaining of p19 or p‐p53 with α‐SMA in ATM ^+/+^ and ATM ^+/−^ mice after MI. G, Representative images and statistical analysis of SA‐β‐gal staining in isolated cardiac fibroblasts after hypoxia/oxygenation treatment and (H) cardiac fibroblasts from ATM ^+/+^ and ATM ^+/−^ mice after MI surgery. n=4 in each group. *P<0.05 vs the corresponding ATM ^+/−^ group. Bar=50 μm.

Increased cardiac fibrosis causes an imbalance between the synthesis and degradation of collagen after MI, which is predominantly regulated by myofibroblasts.^3^ We first examined myofibroblast accumulation and collagen synthesis. The mRNA levels of α‐SMA, collagen I, and collagen III were determined by quantitative real‐time PCR. As shown in Figure 5A, MI evoked α‐SMA, collagen I, and collagen III upregulation, but the mRNA levels of α‐SMA and collagen I were markedly elevated in ATM^+/−^ mice compared with those in WT mice at day 7 after MI. Western blot analysis for α‐SMA and immunostaining of collagen I were further performed, confirming that the protein levels of α‐SMA and collagen I were increased in MI‐injured ATM^+/−^ mice compared with those in the WT group (Figure 5B and 5C).

It has been reported^8^, ^32^ that fibroblast senescence is involved in multiple‐organ fibrosis, including the liver, heart, and skin, and our previous study demonstrated that MI induces fibroblast senescence, contributing to excessive fibrosis.^8^ Therefore, we examined the senescence‐related protein level and fibroblast senescence by using SA‐β‐gal as a marker. Western blotting analysis showed that ATM haplodeficiency lowered p21 expression in infarcted heart compared with that in ATM^+/+^ heart (Figure 5D). SA‐β‐gal staining also demonstrated that there were more SA‐β‐gal^+^ cells in infarcted WT mouse heart compared with that in ATM^+/−^ mice (Figure 5E). Furthermore, costaining of α‐SMA and p19/phosphor‐p53 were also performed to show that there were more double positive cells accumulated in WT mouse heart than that in ATM^+/−^ mice 7 days after MI (Figure 5F), which indicated that ATM haplodeficiency decreased cardiac fibroblast senescence. Furthermore, we isolated the cardiac fibroblast from both WT and ATM^+/−^ mouse heart after MI surgery, and SA‐β‐gal staining showed there were fewer positive cells in ATM^+/−^ fibroblasts (Figure 5G). The similar result was also presented in neonatal cardiac fibroblast from WT and ATM^+/−^ mice in response to hypoxia/reoxygenation stimulation (Figure 5H).

### ATM Haplodeficiency Suppresses Post‐MI Angiogenesis

**Figure 6:**
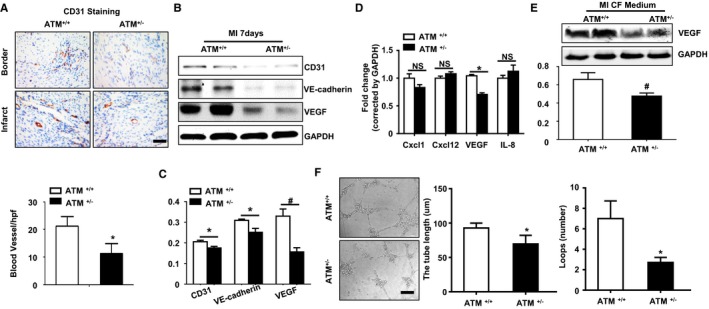
Ataxia telangiectasia mutated (ATM) haplodeficiency suppresses post–myocardial infarction (MI) angiogenesis. A, Immunohistochemical staining and bar graph showing CD31 expression and angiogenesis at day 28 in the hearts of ATM ^+/+^ and ATM ^+/−^ mice post MI. B, Western blot analysis (C) showed CD31, vascular endothelial cadherin (VE‐cadherin), and vascular endothelial growth factor (VEGF) expressions in the hearts of ATM ^+/+^ and ATM ^+/−^ mice at day 7 post MI. D, Quantitative real‐time polymerase chain reaction analysis showed the mRNA levels of chemokine (C‐X‐C motif) ligand (Cxcl) 1, Cxcl12, VEGF, and Cxcl8 in isolated cardiac fibroblasts (CFs) from ATM ^+/+^ and ATM ^+/−^ mice after MI surgery. E, Western blot analysis of VEGF expression in cultured medium of isolated CFs from ATM ^+/+^ and ATM ^+/−^ mice after MI surgery. F, Representative images and bar graph showing tube formation capacity of human umbilical vein endothelial cells on matrigel with stimulation of conditional culture medium from isolated CFs of ATM ^+/+^ and ATM ^+/−^ mice after MI surgery. n=5 in each group. *P<0.05 vs corresponding ATM ^+/+^ group. Bar=50 μm. ^#^ *P*<0.01. IL indicates interleukin; NS, not significant.

As one of the key events post MI that defines post‐MI outcomes, angiogenesis was examined. Immunostaining of CD31 at day 28 post MI showed that ATM haplodeficiency decreased capillary number (Figure 6A). Results from Western blot analysis showed that CD31, VE‐cadherin, and VEGF expression was decreased in ATM haplodeficient heart at day 7 post MI (Figure 6B and 6C). It is reported that the senescent cells could modulate the angiogenesis through secreted cytokines, including CXCL1, CXCL12, VEGF, and CXCL8, known as SASP. To identify whether the angiogenesis was affected by SASP, we examined the mRNA levels of CXCL1, CXCL12, VEGF, or CXCL8 by real‐time PCR in isolated cardiac fibroblasts post MI. The result showed that VEGF expression was higher in fibroblast from infarcted WT mouse than that in ATM^+/−^ fibroblast, while there was no difference in CXCL1, CXCL12, and CXCL8 expressions between the groups (Figure 6D). Results from Western blot analysis of VEGF in the supernatant of cultured cardiac fibroblast showed similar results (Figure 6E). To further explore the role of SASP in modulating angiogenesis, tube formation of human umbilical vein endothelial cells was performed. As shown in Figure 6F, conditional medium from isolated ATM^+/+^ cardiac fibroblast post MI promoted tube formation of human umbilical vein endothelial cells compared with that of ATM haplodeficiency.

### ATM Haplodeficiency Does Not Affect Inflammatory Cell Infiltration in Post‐MI Heart

A large number of reports have indicated that inflammatory cell influx is a highly sophisticated system that contributes to post‐MI cardiac repair.^1^, ^22^ We then performed flow cytometry analysis in both WT and ATM^+/−^ mouse hearts 7 days after undergoing either sham or MI surgery. As shown in Figure S3, MI significantly increased CD45^+^ cell infiltration in both WT and ATM^+/−^ mouse hearts. Moreover, there was no difference in infiltration of macrophages (CD45^+^F4/80^+^CD11b^+^), T cells (CD45^+^CD3^+^), T‐helper cells (CD45^+^CD3^+^CD4^+^), or cytotoxic T cells (CD45^+^CD3^+^CD8^+^) in both ATM^+/+^ and ATM^+/−^ mouse hearts post MI. However, ATM haplodeficiency increased the infiltration of M2 macrophages (CD45^+^F4/80^+^CD11b^+^CD206^+^) and decreased M1 macrophages (CD45^+^ F4/80^+^ CD11b^+^CD206^−^) in infarcted heart compared with WT mouse heart.

In addition, cytokines, which have been reported to be involved in post‐MI cardiac remodeling^3^, ^33^ were also measured at the indicated time points. Real‐time PCR analysis showed that the mRNA levels of interleukin 1β, interleukin 6, tumor necrosis factor‐α, or transforming growth factor‐β were significantly upregulated after MI, peaked at day 3, and declined at day 7 after MI in WT mice. However, ATM haplodeficiency suppressed the elevation of interleukin 1β, interleukin 6, or transforming growth factor‐β mRNA post MI, while there was no difference in mRNA level at day 3 post MI. Moreover, the trend in tumor necrosis factor‐α level was not altered by ATM haplodeficiency (Figure S4).

## Discussion

In the present study, our results demonstrate that MI induces senescence in an ATM‐dependent pathway mainly in cardiac fibroblast, which not only decreased the excessive collagen expression but also promoted angiogenesis through SASP, preserving cardiac function.

Cellular senescence has been seen as a trigger of tissue remodeling in both embryonic development and tissue damage.^5^ Senescent cells contribute to the elimination of damaged cells, while permanent cell senescence could be detrimental. Here, we showed that the senescence‐related proteins were upregulated both in human specimens and mouse heart in response to MI, and senescent cells were accumulated in mouse heart post MI (Figures 1 and 2). Our results are consistent with a report that senescent stellate cells decreased extracellular matrix degradation and increased immune cells recruitment via SASP, limiting the cirrhosis induced liver fibrosis.^4^

As a regulatory kinase upstream of p53, ATM responds to a variety of stimuli, including telomere dysfunction, mitogenic signals, oxidative stress, and DNA damage. Therefore, we investigated the role of ATM in post‐MI cardiac remodeling and heart failure. It has been reported that ATM deficiency worsens cardiac dysfunction and dilation 7 days after MI mainly through increasing cardiac fibrosis and apoptosis^34^ and delaying the inflammatory response.^35^ Consistent with these reports, our present study also demonstrated that senescence regulator ATM has a protective role in post‐MI heart failure (Figure 3 and Table). As an upstream kinase that senses different stimuli, ATM can be activated by oxidative stress,^17^ DNA double‐strand breaks,^14^ or even transforming growth factor‐β,^36^ and these stimuli are associated with MI‐induced heart failure. MI induces oxidative stress and reactive oxygen species (ROS) generation in the ischemic myocardium, which directly damages cellular components and causes cell death. Moreover, ROS also stimulate inflammatory cytokine production and extracellular matrix degradation, promoting left ventricular dilation and heart failure.^19^, ^37^ It is known that ATM is an important sensor of ROS^17^ that also maintains ROS balance.^38^ Our present study showed that ATM haplodeficiency increases the expansion index and worsened heart failure, which may be attributed to an ROS imbalance after MI (Figures 3 and 4).

Cardiac fibrosis determines the outcome of cardiac function post MI, and myofibroblast accumulation is necessary for fibrosis. Our results showed that in response to MI, ATM haplodeficiency accelerated heart failure and increased cardiac fibrosis as well as fibroblast accumulation (Figure 5). A previous study showed that cardiac fibroblasts underwent senescence after MI, and p53 deficiency decreased fibroblast senescence as well as cardiac fibrosis in response to MI.^8^ In the present study, we further showed that ATM was responsible for MI‐induced fibroblast senescence, as ATM haplodeficiency resulted in increased myofibroblast accumulation and fibrosis by suppressing fibroblast senescence, as shown by reduced SA‐β gal staining and p21 and p19 levels (Figure 5).

Angiogenesis plays an important role in preserving cardiac function post MI. Angiogenesis is indirectly enhanced by chemokines, such as CXCL12, CXCL8, CXCL1, and VEGF.^39^ Senescent cells secreted multiple proteins, known as SASP, which modulates angiogenesis and inflammation. Senescent primary human fibroblast–secreted VEGF explained why senescent cells stimulate tumorigenesis in vivo.^40^ In addition, senescent aged fibroblasts also secreted CCL5 and induced proliferation of prostate epithelial cells and expression of genes that modulate angiogenesis.^41^ ROS‐induced ATM activation is necessary for pathological neoangiogenesis and ATM deficiency in mice blocked pathological neoangiogenesis in the retina.^42^ ATM deficiency also lowered tumor angiogenesis and enhanced the antiangiogenic action of VEGF blockade.^42^ Similar to these study findings, our study found that senescent fibroblast–secreted VEGF promoted human umbilical vein endothelial cell tube formation, and ATM haplodeficiency suppressed fibroblast senescence and inhibited angiogenesis in vitro and in vivo.

## Conclusions

The present study showed the protective role of ATM in post‐MI cardiac fibrosis through regulation of cardiac fibroblast senescence, with the underlying mechanism possibly attributed to elevated VEGF secretion.

## Sources of Funding

This work was supported by the National Natural Science Foundation of China (grant numbers 81570423, 81230006, 81670222, and 81100094), the Beijing Nova Program (Z171100001117019), and the Beijing Natural Science Foundation (7141003).

## Disclosures

None.

## Supporting information


**Data S1.** Supplemental methods.
**Figure S1.** Senescence‐associated gene expression in normal and infarcted human hearts. Immunostaining of senescent‐related proteins (A) ataxia telangiectasia mutated (ATM), (B) p16, (C) p21, and (D) p53 in human normal and myocardial infarction (MI) hearts. The senescence‐related gene expression was examined in an infarcted heart of a transplant recipient (57 years, male, 15 days post MI) by immunohistochemical staining of ATM, p53, p21, and p16, and slides were then scanned using a ScanScope AT turbo (Aperio) to obtain digital pictures. The images show that the senescence‐related proteins were elevated in the infarct area.
**Figure S2.** Ataxia telangiectasia mutated (ATM) haplodeficiency aggravated cardiac dysfunction post myocardial infarction (MI). A, Representative images of 2,3,5‐triphenyltetrazolium chloride (TTC) staining of ATM^+/+^ and ATM^+/−^ mice hearts at 24 hours after MI surgery. B, Representative of fibrosis in noninfarcted area and statistical analysis of the cross‐sectional area of cardiomyocytes. C, Representative Ladewig staining of cardiac sections in ATM^+/+^ and ATM^+/−^ mice after MI surgery. D, Representative terminal deoxynucleotidyl transferase dUTP nick end labeling staining and statistical analyses of cardiac sections in ATM^+/+^ and ATM^+/−^ mice after MI surgery at the indicated time. *P<0.05 vs the ATM^+/+^ MI group. Bars=100 μm. WGA indicates wheat germ agglutinin.
**Figure S3.** Ataxia telangiectasia mutated (ATM) haplodeficiency has no effect on inflammatory cell infiltration in post myocardial infarction (MI) heart. A, Flow cytometry analysis of leukocytes (CD45^+^), macrophages (CD45^+^F4/80^+^CD11b^+^), M1 macrophages (CD45^+^CD11b^+^F4/80+CD206^−^), M2 macrophages (CD45^+^CD11b^+^F4/80^+^CD206^+^), T cells (CD45^+^CD3^+^), T‐helper cells (CD45^+^CD3^+^CD4^+^), or cytotoxic T cells (CD45^+^CD3^+^CD8^+^) were performed in ATM^+/+^ and ATM^+/−^ mouse hearts at 7 days after sham or MI surgery. B, Bar graph shows the percentage of cells in the heart. n=5 in each group. *P<0.05 vs the corresponding sham group, ^#^ *P*<0.05 vs the ATM^+/+^ MI group, not significant (NS). SSC indicates side scatter.
**Figure S4.** Ataxia telangiectasia mutated (ATM) haplodeficiency increased the level of cytokines in post myocardial infarction (MI) heart. A, Quantitative real‐time polymerase chain reaction (PCR) was performed to detect the mRNA level of interleukin (IL)–1β, IL‐6, tumor necrosis factor‐α (TNF‐α), and transforming growth factor‐β (TGF‐β) in the hearts of ATM^+/+^ and ATM^+/−^ mice at the indicated time points post MI. *P<0.05 vs the corresponding sham group; ^#^ *P*<0.01 vs the corresponding sham group; ^†^ *P*<0.05 vs the ATM^+/+^ MI group.
