# Supplementary material for: Haplodeficiency of Ataxia Telangiectasia Mutated Accelerates Heart Failure After Myocardial Infarction
Source: J Am Heart Assoc. 2017 Jul 19;6(7):e006349. doi: 10.1161/JAHA.117.006349 (PMC5586323; doi:10.1161/JAHA.117.006349)
Supplement: Supplementary file 1 — Data S1. Supplemental methods. Figure S1. Senescence‐associated gene expression in normal and infarcted human hearts. Immunostaining of senescent‐related proteins (A) ataxia telangiectasia mutated (ATM), (B) p16, (C) p21, and (D) p53 in human normal and myocardial infarction (MI) hearts. The senescence‐related gene expression was examined in an infarcted heart of a transplant recipient (57 years, male, 15 days post MI) by immunohistochemical staining of ATM, p53, p21, and p16, and slides were then scanned using a ScanScope AT turbo (Aperio) to obtain digital pictures. The images show that the senescence‐related proteins were elevated in the infarct area. Figure S2. Ataxia telangiectasia mutated (ATM) haplodeficiency aggravated cardiac dysfunction post myocardial infarction (MI). A, Representative images of 2,3,5‐triphenyltetrazolium chloride (TTC) staining of ATM+/+ and ATM+/− mice hearts at 24 hours after MI surgery. B, Representative of fibrosis in noninfarcted area and statistical analysis of the cross‐sectional area of cardiomyocytes. C, Representative Ladewig staining of cardiac sections in ATM+/+ and ATM+/− mice after MI surgery. D, Representative terminal deoxynucleotidyl transferase dUTP nick end labeling staining and statistical analyses of cardiac sections in ATM+/+ and ATM+/− mice after MI surgery at the indicated time. *P<0.05 vs the ATM+/+ MI group. Bars=100 μm. WGA indicates wheat germ agglutinin. Figure S3. Ataxia telangiectasia mutated (ATM) haplodeficiency has no effect on inflammatory cell infiltration in post myocardial infarction (MI) heart. A, Flow cytometry analysis of leukocytes (CD45+), macrophages (CD45+F4/80+CD11b+), M1 macrophages (CD45+CD11b+F4/80+CD206−), M2 macrophages (CD45+CD11b+F4/80+CD206+), T cells (CD45+CD3+), T‐helper cells (CD45+CD3+CD4+), or cytotoxic T cells (CD45+CD3+CD8+) were performed in ATM+/+ and ATM+/− mouse hearts at 7 days after sham or MI surgery. B, Bar graph shows the percentage of cells in the heart. n=5 in each g [file JAH3-6-e006349-s001.pdf]

SUPPLEMENTAL MATERIAL

Data S1.

Supplemental Methods

Human specimens

Human MI heart was obtained from one myocardial infarction patient undergoing heart transplantation surgery in Beijing Anzhen Hospital who signed informed consent. The normal human heart was obtained from heart transplantation donors. The study was done in accordance with the Declaration of Helsinki and approved by the Institutional Review Board of Beijing Anzhen Hospital, Capital Medical University. Patients provided a written consent.

Histological analysis

To measure the infarct size, TTC staining was performed as described[1]. Mice were anesthetized with 1% sodium pentobarbital (100 mg/kg i.p.). The heart was excised and the ascending aorta was cannulated (distal to the sinus of Valsalva), then perfused retrogradely with 1% 2,3,5-triphenyl-tetrazolium chloride and incubated for 15 min at 37°C to visualize infarcted region. Then, the heart was frozen at −80 °C for 10 min and cut into slices (5–6 slices/heart).

Wheat germ agglutinin (WGA) staining was performed on deparaffinized heart sections with 100-μg/mL FITC-labeled WGA (Sigma-Aldrich, St. Louis, MO) for 90 min to evaluate the cross-sectional cardiomyocyte area. At least 40 cells per slide were measured to calculate the cell area as we described previously[2].

For Ladewig staining, sections were brought to water with xylene and ethanol and treated with Ladewig's and Weigert's solution[3].

Flow cytometry analysis

The inflammatory cells infiltration in sham or post MI mouse hearts were analyzed by flow cytometry as described before[4]. In brief, fresh hearts were minced and digested with 1.6 mg/ml collagenase IA (Sigma Aldrich, Tokyo, Japan) and 200 μg/ml DNase I (Roche, Indianapolis, IN, USA) in PBS at 37 °C for 50 min. Flow cytometry was carried out using the following antibodies: PerCP-Cy5.5 anti-mouse CD45.2, PE anti-mouse F4/80 (Biolegend, San Diego, CA, USA), Alexa APC anti-mouse CD11b, FITC anti-mouse CD206, PE anti-mouse CD3e, APC anti-mouse CD4, APC-cy7 anti-mouse CD8 (all from BD Biosciences). Flow cytometry data were acquired using BD LSRFortessa (BD Biosciences) and analyzed by BD FACSDiva software (BD Biosciences)

RNA extraction and real-time PCR analysis

Total RNAs were extracted using the Trizol method according to the manufacturer's protocol (Invitrogen, Carlsbad, CA). Two micrograms of purified RNA was used for cDNA synthesis. Reverse transcription of mRNA was accomplished by 2x SYBR master mix (Takara, Otsu, Shiga), using a BIO-RAD iCycler iQ5 (Bio-Rad, Hercules, CA). Primers used in this study were as follows: IL-1β, forward 5′-CTTCAGGCAGGCAGTATCACTCAT-3′ and reverse 5′-TCTAATGGGAACGTCACACACCAG-3′; IL-6, forward 5′-CTTCCATCCAGTTGCCTTCTTG-3′ and reverse 5′-AATTAAGCCTCCGACTT GTGAAG-3′; TNF-α, forward 5′-GCCACCACGCTCTTCTGTCT-3′ and reverse 5′-GTCTGGGCCA TGGAACTGAT-3′; TGF-β, forward 5′-CAACAATTCCTGGCGTTACCTTGG-3′ and reverse 5′ GAAAGCCCTGTATTCGTCTCCTT-3′. Real-time PCR was carried out with the use of the Bio-Rad iQ5 (Hercules, CA, USA).

Figure S1.

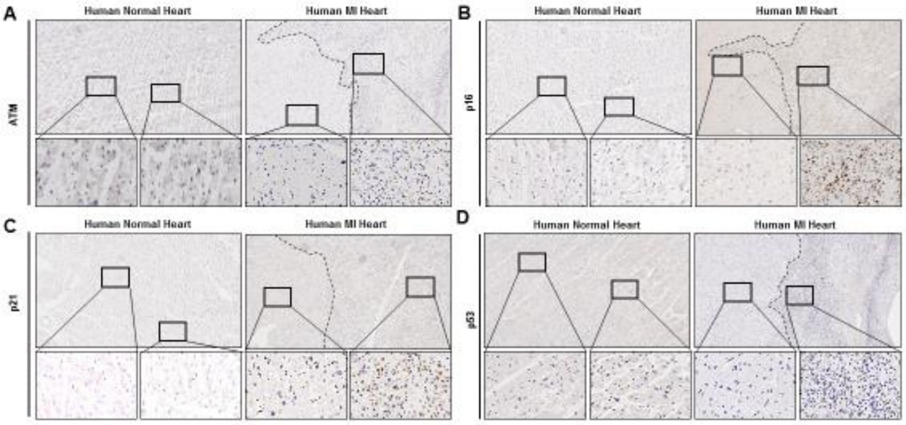

A
Human Normal Heart
Human MI Heart
ATM
B
Human Normal Heart
Human MI Heart
p16
C
Human Normal Heart
Human MI Heart
p21
D
Human Normal Heart
Human MI Heart
p53

Figure S2.

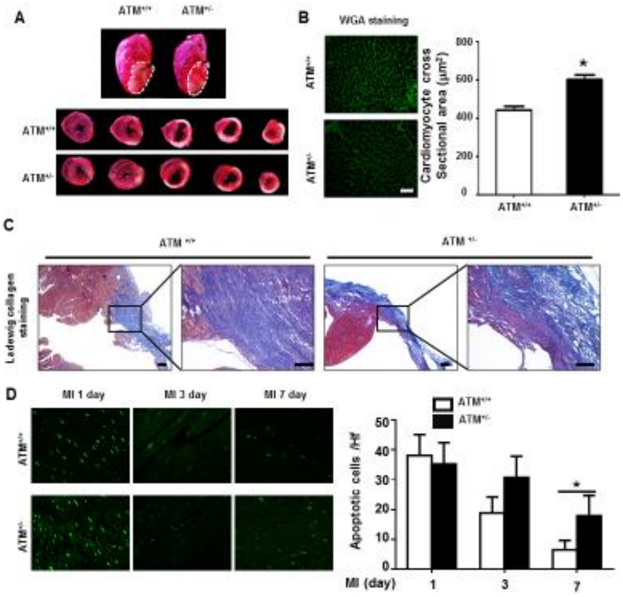

A
ATM+/+
ATM+/-
ATM+/+
ATM+/-
B
WGA staining
ATM+/+
ATM+/-
Cardiomyocyte cross Sectional area (μm²)
800
600
400
200
0
*
ATM+/+
ATM+/-
C
ATM +/+
ATM +/-
Ladewig collagen staining
D
MI 1 day
MI 3 day
MI 7 day
ATM+/+
ATM+/-
Apoptotic cells /Hf
50
40
30
20
10
0
*
MI (day)
1
3
7

Figure S3.

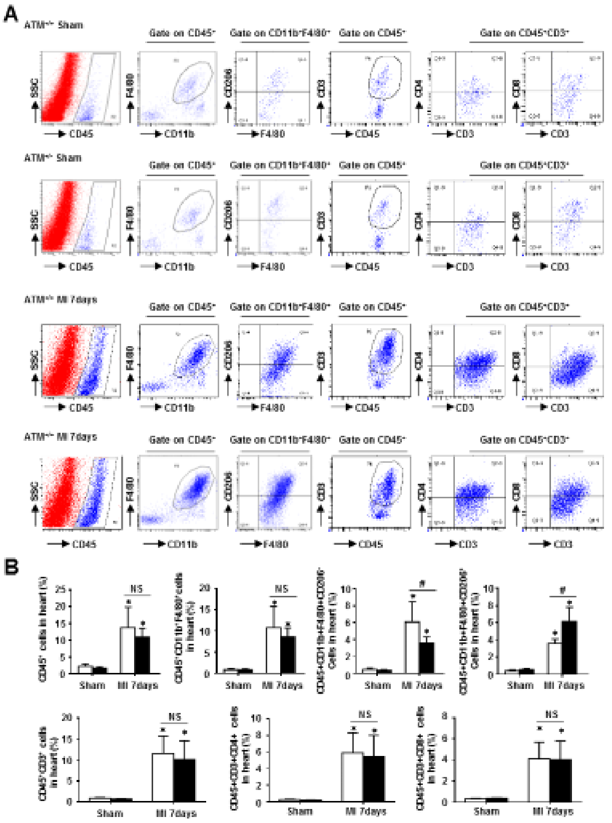

Figure S4.

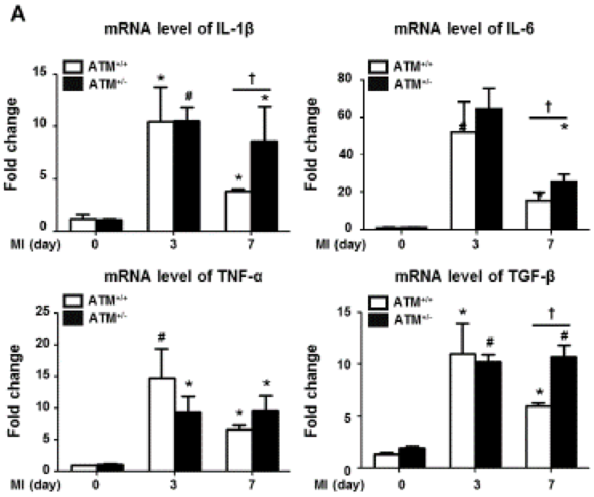
A
mRNA level of IL-1β
mRNA level of IL-6
mRNA level of TNF-α
mRNA level of TGF-β
ATM+/+
ATM+/-
Fold change
MI (day)
0
3
7

Supplemental Figure Legends:

Figure S1. Senescence associated genes expression in normal and infarcted human heart.

Immunostaining of senescent related proteins **A)** ATM, **B)** p16, **C)** p21 and **D)** p53 in human normal heart and MI hearts. The senescence related genes expression was examined in a heart of transplant recipient of infarcted patient (57 years, male, 15 days post MI) by immunohistochemical staining of ATM, p53, p21 and p16, and slides were then scanned by using a ScanScope AT turbo (Aperio, Vista, CA) to obtain digital pictures. The images showed that the senescence related proteins were elevated in the infarct area. ATM: Ataxia telangiectasia mutated.

Figure S2. ATM haplodeficiency aggravated cardiac dysfunction post MI.

A) Representative images of TTC staining of $ATM^{+/+}$ and $ATM^{+/-}$ mice hearts at 24 hours after MI surgery. **B)** Representative of fibrosis in no-infarcted area and statistical analysis of the cross-sectional area of cardiomyocytes. **C)** Representative Ladewig staining of cardiac sections in $ATM^{+/+}$ and $ATM^{+/-}$ mice after MI surgery. D) Representative TUNEL staining and statistical analyses of cardiac sections in $ATM^{+/+}$ and $ATM^{+/-}$ mice after MI surgery at the indicated time. *$p<0.05$ vs. $ATM^{+/+}$ MI group. Bars=100μm. ATM: Ataxia telangiectasia mutated; MI: Myocardial infarction; TTC: 2,3,5-triphenyltetrazolium chloride; WGA: Wheat germ agglutinin.

Figure S3. ATM haplodeficiency has no effect on inflammatory cells infiltration in post MI heart

A) Flow cytometry analysis of leukocytes ($CD45^+$), macrophages ($CD45^+F4/80^+CD11b^+$), M1 macrophages ($CD45^+CD11b^+F4/80^+CD206^-$), M2 macrophages ($CD45^+CD11b^+F4/80^+CD206^+$), T cells

($CD45^+CD3^+$), T helper cells ($CD45^+CD3^+CD4^+$) or cytotoxic T cells ($CD45^+CD3^+CD8^+$) were performed in $ATM^{+/+}$ and $ATM^{+/-}$ mice hearts at 7 days after sham or MI surgery. **B)** Bar graph shows the percentage of cells in the heart. n= 5 in each group, *p<0.05 *vs.* corresponding sham group, #p<0.05 *vs.* $ATM^{+/+}$ MI group, ns means not significant. ATM: Ataxia telangiectasia mutated; MI: Myocardial infarction; SSC: Sidescatter.

Figure S4. ATM haplodeficiency increased the level of cytokines in post MI heart

A) Quantitative real-time PCR was performed to detect the mRNA level of IL-1β, IL-6, TNF-α, and TGF-β in the hearts of $ATM^{+/+}$ and $ATM^{+/-}$ mice at the indicated time points post MI. *P<0.05 *vs.* corresponding sham group; $^{\#}$P<0.01 *vs.* corresponding sham group; †P<0.05 *vs.* $ATM^{+/+}$ MI group. ATM: Ataxia telangiectasia mutated; MI: Myocardial infarction; IL-1β: Interleukin-1 β; IL-6: Interleukin-6; TNF-α: Tumor necrosis factor-α; TGF-β: Transforming growth factor-β.

Supplemental References:

1. Zhang T, Zhang Y, Cui M, Jin L, Wang Y, Lv F, Liu Y, Zheng W, Shang H, Zhang J, Zhang M, Wu H, Guo J, Zhang X, Hu X, Cao CM, Xiao RP. CaMKII is a RIP3 substrate mediating ischemia- and oxidative stress-induced myocardial necroptosis. *Nat Med.* 2016;22:175-182.

2. Yang M, Zheng J, Miao Y, Wang Y, Cui W, Guo J, Qiu S, Han Y, Jia L, Li H, Cheng J, Du J. Serum-glucocorticoid regulated kinase 1 regulates alternatively activated macrophage polarization contributing to angiotensin II-induced inflammation and cardiac fibrosis. *Arterioscler Thromb Vasc Biol.* 2012;32:1675-1686.

3. Al-Sawaf O, Fragoulis A, Rosen C, Kan YW, Sonmez TT, Pufe T, Wruck CJ. Nrf2 protects against TWEAK-mediated skeletal muscle wasting. *Sci Rep.* 2014;4:3625.

4. Li TT, Jia LX, Zhang WM, Li XY, Zhang J, Li YL, Li HH, Qi YF, Du J. Endoplasmic reticulum stress in bone marrow-derived cells prevents acute cardiac inflammation and injury in response to angiotensin II. *Cell Death Dis.* 2016;7:e2258.
